# Clinical Cases

**Published:** 1881-07

**Authors:** John F. Miller

**Affiliations:** Newark, N. J.


### CLINICAL CASES.

JOHN F. MILLER, M. D., NEWARK, N. J.

### PROLAPSE OF HÆMORRHOIDAL TUMOR AND RECTUM.

MR. G. Age seventy-three; large, obese, florid complexion; after two or three days of general indisposition, the tumor made its appearance; not after stool. On second day found a tumor the size of double fist, very hard, hot and purplish color; complained of much pain, throbbing, with sense of constriction; scanty black stools; frequent ineffectual efforts to urinate; pulse 60. *Mur. ac.* 90 m., one dose.

The next day, December 18th, much the same. *Mur. ac.* 90 m., one dose.

December 19th. Tongue dry, dark-red, restless prostration, stitching pain in tumor. *Ars.* 200th every three hours.

December 19th, 9 P. M. Tongue moist. *Sac lac.*

December 20th. Very much worse; tumor very blue and painful; tongue dry, red; dizzy; great prostration and distress. *Lach.* 200th every two hours, and asked for counsel.

December 20th, 5 P. M. Dr. Lippe, of New York, saw the case with me and diagnosed *Lach.* As patient had received two doses and seemed a little better, Dr. L. advised *Sac. lac.* as long as improvement continues. Gave rather an unfavorable prognosis, and suggested the c. m. potency of the same remedy if he got worse.

December 21st. A little better; not so much urging to urinate; can pass flates downwards, the first time for years. Pulse 52; less beating in tumor; feeling generally more comfortable. *Sac. lac.*

December 22d. Tumor smaller, and not so dark. *Sac. lac.*

December 23d. Pulse 68; tongue dry; lip catches on teeth. *Sac. lac.*

December 24th. Small stool, without any trouble; tumor shrunken, flabby; tongue dry, but not so red. *Sac. lac.*

December 27th, 10 A. M. Pulse 72, full hard; tongue dry; dark on tip, and in centre; restless, distressed. *Lach.* c. m., one dose.

December 27th, 9 P. M. Severe chill in the afternoon; face very red; thirsty; headache. Pulse 80 full; tumor blue and hard, although only about half of its former size; tongue trembling, hard to protrude, catching on tip; exhaustion seemed profound. *Lach.* c. m., every three hours.

December 28th. Pulse 60; improving. *Sac. lac.*

January 4th. Occasional fair stools; tumor size of butternut; pain in tumor after stools; eczema of legs. *Graph.* c. m., one dose.

January 5th. But little pain in tumor during or after stool; the pain had lasted three or four hours after stool the day before. *Sac. lac.*

January 12th. No pain in tumor, which is size of small butternut; appetite good; bowels regular.

Dr. E. C. Franklin in the transactions of the American Institute, says: "This disease (prolapsus of rectum) is an affection of childhood, but occasionally occurs in old age, when it assumes a serious aspect.

In a case of the latter kind, that came under my observation, the descent of the bowel was so great, as to produce death by consequent derangement and irritation of the system (exhaustion?) It was reduced three times, but so great was the propensity to extrusion that not even a T bandage would retain it in position. Medicines effected little or no good, and the patient sank from exhaustion." What medicines were given, he does not state.

In my case, neighbors and friends thought local treatment should be used. I explained to them, that the prolapsed tumor was not the cause of this sickness, but that his exhaustion and illness was the cause of the tumor, and any local treatment, would be directed to the effect, and not the cause. The treatment would have been at the wrong end, in more than one sense.

After his recovery, the patient said to me, "Do you know that if I had died, during this sickness, without any local treatment, and only one or two slight, insufficient stools in over two weeks, you would have been blamed, unjustly, perhaps; but still it would have injured you very much. Would it not have been politic to have done something to allay the prejudices of the people?"

My answer was, that that line of conduct—the desire to allay allopathic prejudices, was the reason that there were so many mongrels.

Too many so-called homœopathic physicians will sacrifice their homœopathic principles every day to placate allopathic prejudices. Instead of endeavoring to educate people in right principles, they weakly yield to the majority; seemingly justifying the assertion of the old school, that homœopathic physicians only treated trivial cases, that would get well without any medicine, with homœopathic remedies, while serious cases were treated allopathically. This is true of weak-kneed homœopaths, but able homœopaths, under all and every circumstance, adhere rigidly to strict homœopathy as giving always the best results.

Nothing but pure homœopathy saved this man's life. Any other treatment would have assuredly caused death.

DIPHTHERIA, *Lac. can.*—May 8th, 1881. Miss Nellie P., æt. 20. Saw this case at 11 A. M., and obtained the following history: The afternoon of the day previous, began to feel ill and very nervous. A constant dread; a feeling that she was going to become unconscious. Throat a little sore. During the night very restless, awaking often frightened, and not knowing where she was; in the morning, so much prostrated that *she could not turn in bed;* her mother and sister were obliged to turn her. I found her face indicating great anxiety. Complained of some headache and backache, and *so tired.* Pulse 120. Said throat was sore some. On inspecting the throat, I found but little swelling; tonsils very little enlarged, but I saw on both sides of the throat the best illustration of diphtheritic deposit I ever met. On an inflamed red base, three-quarters of an inch long, one quarter inch wide, was the membrane, one-eighth of an inch thick and the same length and width as the base. The anterior edge was of a dirty yellow; the centre more organized, pearly, glistening, white-like cartilage. It was the worst looking throat I ever saw in a practice of sixteen years, for the length of time of forming. I said to the patient, which side of your throat is sore? She answered, the left side now; last night I felt it most on the right side. The right side did seem more firm and dense, and was later in disappearing. I at once put on her tongue, dry, one dose *Lac. can.*, c. m. (Fincke), and *Sac. lac.* in water every two hours.

May 9th. Called at the same hour; found my patient looking bright and cheerful. She asked, after saying that she was better, "Doctor, did you give that dry powder to make me sleep. I went to sleep in a few minutes after you left; had a long nap; woke up feeling so refreshed, and have felt better ever since." The pearly white color had disappeared. The membrane looked as if it could be easily brushed off. Pulse 80. Better in every way, except prostration, which remained about the same. *Sac. lac.*, every three hours.

May 10th. The throat perfectly clear of membrane, leaving a very red, angry base. The prostration not quite so marked. *Sac. lac.*

May 11th. Prostration less. A little appetite. *Sac. lac.*

May 12th. Sitting up; well, except she felt she had been very sick. Dismissed the case.

Requested a mongrel to see this case on the second morning. He thought it was a case of *follicular tonsilitis*, although there was no swelling of the tonsils. Accounted for the *prostration by saying that she was nervous.* He would rather have sworn that it was a case of cancer of the womb, than admit that one dose of *Lac. can.*, c. m., had cured a case of diphtheria in twenty-four hours.

